# The diagnostic/prognostic roles and biological function of the IFIT family members in acute myeloid leukemia

**DOI:** 10.1186/s12920-023-01735-0

**Published:** 2023-11-18

**Authors:** YiFan Zhao, Yi Zhang, WenYi Lu, Rui Sun, RuiTing Guo, XinPing Cao, Xingzhong Liu, Cuicui Lyu, MingFeng Zhao

**Affiliations:** 1 First Center Clinic College of Tianjin Medical University, Tianjin, People’s Republic of China; 2 Department of Hematology, Tianjin First Central Hospital, Tianjin, People’s Republic of China; 3 School of Medicine, Nankai University, Tianjin, People’s Republic of China; 4 State Key Laboratory of Medicinal Chemical Biology, Key Laboratory of Molecular Microbiology and Technology of the Ministry of Education, Department of Microbiology, College of Life Sciences, Nankai University, Tianjin, People’s Republic of China

**Keywords:** Acute myeloid leukemia, IFIT, Biomarker, Prognosis, Immune infiltration, Immune checkpoints, Drug sensitivity

## Abstract

**Background:**

The Interferon-induced protein with tetratricopeptide repeat (IFIT) family, IFIT1/2/3/5, play an important role in different tumors progression. However, the prognosis significance and biological role of IFIT family members in acute myeloid leukemia (AML) remains unclear.

**Methods:**

We obtained the gene expression data and clinical information of 173 AML patients from The Cancer Genome Atlas (TCGA) database. Several databases were used in our study, including GEPIA, MethSurv, STRING, GSCA and GeneMANIA database.

**Results:**

The mRNA expression of IFIT1/2/3/5 was elevated in AML patients and had a high ability to distinguish AML from controls based on the receiver operating characteristic (ROC) curve (AUC > 0.9). Kaplan–Meier survival analysis showed that higher levels of IFIT2/3/5 expression predict poor prognosis in AML patients. Besides, the DNA methylation analysis suggested that 7 CpG sites of IFIT2, 4 CpG sites of IFIT3 and 10 CpG sites of IFIT5 were significantly associated with the prognosis of AML patients. In addition, IFIT2/3/5 expression was significantly positively associated with the immune cell infiltration and immune checkpoint expression, such as CTLA4, PDCD1, LAG3, and TIGIT. Finally, drug sensitivity analysis revealed that AML patients with high expression of IFIT2/3/5 were resistant to multiple drugs, but sensitive to dasatinib.

**Conclusion:**

IFIT family genes might serve as biomarkers for diagnosis, prognosis and drug sensitivity in AML patients. The activation or blocking of IFIT-related signaling pathways may provide novel insights into immunotherapy for patients with AML.

**Supplementary Information:**

The online version contains supplementary material available at 10.1186/s12920-023-01735-0.

## Introduction

Acute myeloid leukemia (AML) is the most common hematologic malignant among adults, which incidence was 3.4–4.3 per 100,000 persons per year in countries such as the United States, United Kingdom, Canada or Australia [1]. AML mainly originated from the abnormal proliferation of immature myeloid cells, complicated with clinical symptoms such as infection, anemia and hemorrhage [2]. Although the understanding of the biological characteristics of AML is deepening, the treatment for AML patients, especially non-acute promyelocytic leukemia(non-APL), still lacks progress, which still dominated by traditional chemotherapy regimens [3]. The remission rate of conventional induction therapy, comprising anthracycline and cytarabine, ranges from 60–85% in patients below the age of 60, whereas it varies between 40–60% in patients aged over 60 years old [4]. Novel targeted drugs like Venetoclax have demonstrated the potential to elevate the complete remission rate beyond 90% [5]. However, the recurrence rate of AML patients has reached 60–80% and the current 5-year survival rate of AML patients is only 26% [6]. Therefore, it is necessary to identify novel prognostic biomarkers which could be helpful for monitoring patients’ prognosis and revealing the underlying molecular mechanisms of AML.

Interferon (IFN) is a group of signaling proteins that synthesized and secreted by body cells, with biological functions such as antiviral infection, inhibitory cell proliferation, anti-tumor metastasis and immune regulation [7–9]. IFN stimulates immune surveillance and cancer immune editing capacity are important parts of its antitumor effect [8]. All IFNs are secreted ligands of surface receptors in cell-membrane and regulate the expression of hundreds of interferon stimulated genes (ISGs) [10]. Among them, the interferon-induced protein with tetratricopeptide repeats(IFIT) family is thought of as one of the most highly-responsive ISGs, which includes 4 members: IFIT1, IFIT2, IFIT3 and IFIT5 [11]. IFIT family encodes cytoplasmic proteins which size are between 47-56 kDa, and these proteins are characterized by the special tetratricopeptide repeat helix-rum-helix (TPR) [12]. The TPR motif is a protein–protein interaction module and exercises various functions such as translation initiation, double-stranded RNA recognition and participation in cell migration and proliferation [13, 14]. It is reported that the proapoptotic effect of IFIT family may contribute to the antiviral and antiproliferative functions of IFN through TPR motif [15].

Increasing evidence indicates that the IFIT family is associated with the tumor initiation, progression and invasion in many cancer types, such as gastric carcinoma, colon cancer, bladder cancer, pancreatic cancer and oral squamous cell carcinoma [16–19]. However, the prognosis significance and biological function of IFIT family in AML is largely unknown. With the development of high-throughput sequencing and gene chip detection, the attention to the molecular mechanism of AML is rising. These molecules pushed the diagnosis and prognostic evaluation into a new era, and targeted drugs based on the different targets of the pathogenesis provided some new therapeutic choices for AML patients. In the present study, we carried out a comprehensive analysis of the expression, diagnosis and prognosis values of the IFIT family in AML. Additionally, we conducted Cox regression analysis to evaluate the predictive significance of IFIT family expression in combination with well-established adverse prognostic factors, including advanced age, elevated white blood cell count, complex cytogenetics, FLT3 mutation, NPM1 mutation, and RAS mutation [20]. Furthermore, multiple methods such as functional enrichment, immune infiltration, immune checkpoints analysis were employed to investigate the potential mechanisms of IFIT family in AML. This research may be helpful to predict clinical outcomes for AML patients and provide novel insights into the molecular mechanism of AML progression.

## Materials and methods

### Data sources

The Cancer Genome Atlas (TCGA) is a large-scale cancer genomics research project conducted in the United States that collects multiple omics data on more than 30 types of tumors. First, we acquired the data on gene expression and detailed clinical information of 173 AML patients from TCGA official website (https://www.cancer.gov/tcga). The clinical information included age, gender, cytogenetic risk, FLT3 mutation, NPM1 mutation, RAS mutation status. Secondly, the RNA sequencing data of 70 normal blood cases were accessed from the Genotype-Tissue Expression (GTEx) database (http://www.gtexportal.org). “Bioconductor” R package (version 3.10) was used for data preprocessing, and the “limma” R (version 3.10) package was used to correct and normalize the data.

### Gene expression analysis

As a first step, we assessed the mRNA expression of IFITs in multiple human tissues using the "GTEx Expression" module in the GSCA database (http://bioinfo.life.hust.edu.cn/GSCA/). Next, the pan-cancer analysis was performed to compare the expression difference of IFIT1/2/3/5 between 33 types of human tumors and corresponding healthy tissues through TCGA datasets. Subsequently, the expression difference of IFIT1/2/3/5 between AML tumor samples (*n* = 173) and control blood samples (*n* = 70) was further validated through the “expression on box plots module” of Gene Expression Profiling Interactive Analysis (GEPIA) database (version 1.0; http://gepia.cancer-pku.cn/index.html). Statistical significance was determined by *P* < 0.05.

### Interaction network analysis of IFIT family

A spearman analysis was performed using TCGA data to evaluate the correlation among members of the IFIT family members. *P* < 0.05 indicated statistical significance. Furthermore, the STRING database (https://cn.string-db.org/) was applied in order to construct a protein–protein interaction (PPI) network for the IFIT family. Additionally, we used the GeneMANIA database (http://genemania.org/) to develop the gene network of the IFIT family and predicted potential target genes.

### Diagnostic and prognostic value of IFIT family

After that, we employed the “pROC” R packages (version 1.18.4) to evaluate the diagnostic performance of IFIT family members in discriminating AML samples from normal blood cases. Diagnostic effectiveness could be summarized using the area under the curve (AUC) of the receiver operating characteristic curve (ROC). Regarding to prognostic significance, we first performed the univariate Cox regression analysis with IFITs mRNA expression and clinical features, including age, gender, cytogenetic risk, FLT3 mutation status, NPM1 mutation status, RAS mutation status, WBC count (× 10^9^/L). The Hazard ratio (HR) and 95% confidence interval (CI) were analyzed for evaluating the correlation between IFITs expression level and mortality of AML patients. Subsequently, we analysis the overall survival for AML patients according to the IFITs high expression and IFITs low-expression groups through the Kaplan–Meier curve. Briefly, patients are stratified into high- or low-expression groups according to the median level of IFITs expression. Finally, the “Regression Modeling Strategies(rms)” and “Survival” R package (version 5.1.4) were used to construct the prognostic nomogram model to predict the overall survival of AML patients.

### DNA methylation analysis

We downloaded the DNA methylation map of IFIT2, IFIT3 and IFIT5 in AML from “Gene visualization” module of MethSurv database by choosing “Acute Myeloid Leukemia [AML] TCGA March 2017” in “Cancer”. The relationship between methylation level of IFITs and survival of patients with AML was also analyzed by MethSurv database (https://biit.cs.ut.ee/methsurv/).

### Functional enrichment analysis

First of all, we identified the top 1000 genes positively correlated to IFIT2/3/5 by Spearman correlation analysis according to TCGA data. According, a total of 404 co-correlated genes of IFIT2/3/5 were screened out through veen map. To elucidate the biological pathways and functions of IFITs in AML, the Kyoto Encyclopedia of Genes and Genomes (KEGG) enrichment analysis [21, 22] for these co-correlated genes were performed by “ClusterProfiler” R package (version 4.0.5).

### Immune-related analysis

Firstly, we used ssGSEA algorithm in the “GSVA” R package (version 1.40.1) to calculate the infiltration level of 24 types immune cells in each tumor sample according to the reported biomarkers of immune cells [23]. Subsequently, the Spearman correlation analysis between ACE2 expression and tumor infiltration levels in AML was performed. Also, we divided the AML patients into the high IFIT expression groups and the low expression groups and analyzed the difference of immune cells infiltration level between the two groups. The association of IFITs and immune checkpoints in AML was also assessed through Spearman correlation analysis, including PDCD1 (PD1), BTLA, CTLA4, CD274 (PD-L1), LAG3, TIGIT, KLRC1, ICOS and HAVCR2 (TIM-3). *P* < 0.05 was considered statistically significant.

### Drug sensitivity analysis

We downloaded the IC50 of 481 small molecules and its corresponding mRNA gene expression from Cancer Therapeutics Response Portal (CTRP) database (version 2.1; https://portals.broadinstitute.org/ctrp.v2.1/), and the correlation between IFIT2, IFIT3 and IFIT5 expression and drug IC50 was performed by Pearson correlation analysis through the module of “Drug” in GSCA database. *P*-value was adjusted by false discovery rate (FDR).

## Results

### Expression of IFIT genes in AML

**Fig. 1:**
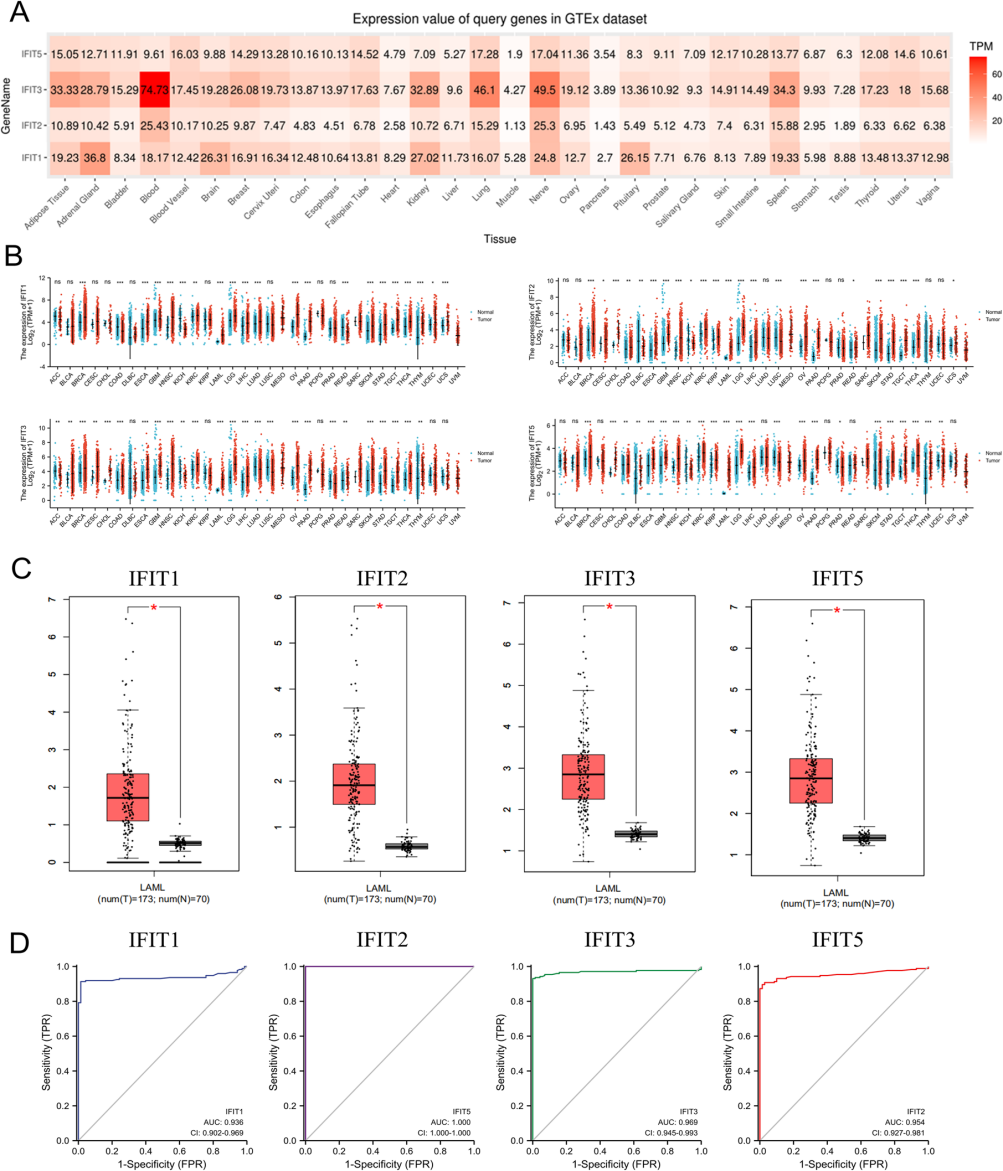
The expression analysis of IFIT family members. **A** Heatmap of the expression profiles of IFIT members in human normal tissues based on GTEx dataset. **B** The expressing levels of IFIT members across diverse cancer types. **C** Analysis of TRPV1/2/3/5 expression in AML and adjacent normal tissues through GEPIA database. **D **The ROC curve of IFITs mRNA expression in AML and normal tissues. *P < 0.05, * *P < 0.01, ***P < 0.001

First, we mined the GTEx database to investigate the expression distribution of IFIT family in human normal tissues. As shown in Fig. 1A, IFIT2 and IFIT3 are expressed at higher levels in human blood tissues than other tissues, while the expression of IFIT1 in adrenal gland and kidney was obviously upregulated, suggesting that the expression of IFIT family genes is tissue-specific (Fig. 1A). Subsequently, the expression difference of IFIT1/2/3/5 between human tumors and normal tissues was compared through TCGA datasets. Among 33 types of human cancers, IFIT1, IFIT2, IFIT3, IFIT5 were abnormally expressed in 21, 23, 24 and 23 cancer types respectively (Fig. 1B). Using GEPIA database, we found that all four IFIT family members were significantly upregulated in AML tumor samples(*n* = 173) compared with control blood samples (*n* = 70) (Fig. 1C). Importantly, the ROC curve showed that the AUC of IFIT1/2/3/5 in AML was 0.936,1.000,0.969 and 0.954 respectively. Given the high ability of IFITs to discriminate AML from normal tissues (AUC > 0.9), IFIT family genes are promising diagnostic markers for AML (Fig. 1D).

### Gene and protein network of IFIT family members

**Fig. 2:**
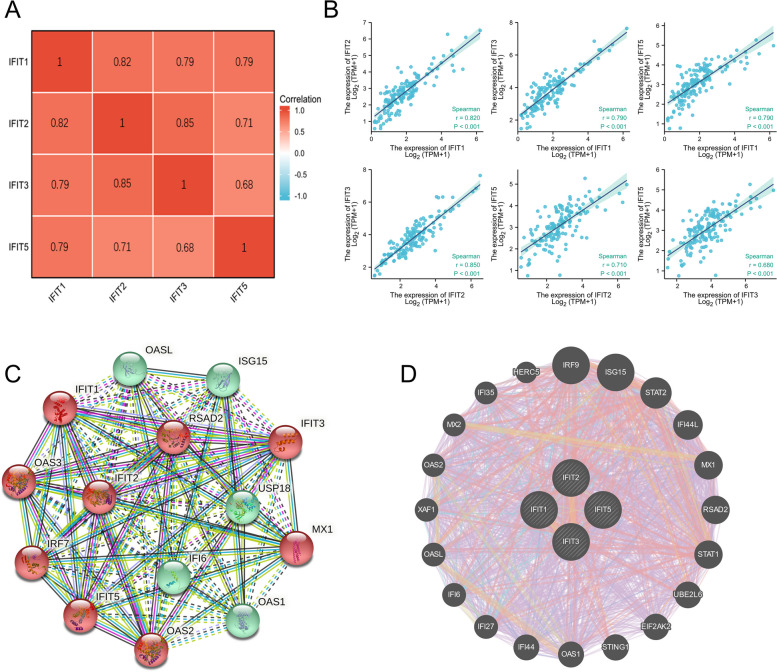
The correlation analysis among IFIT family members. **A** The heat map and **B** the scatter plots of the correlation among IFIT1/2/3/5. **C** A network diagram of interactions between proteins encoded by genes of the IFIT family. **D** The gene network associated with the IFIT family analyzed by GeneMANIA database

We then explored the expression correlation among IFIT family genes in LAML through Spearman’s correlation analysis (Fig. 2A-B). As a result, there was a significantly positive correlation among four IFIT family members in AML. For instance, IFIT1 expression positively correlated with IFIT2 (*r* = 0.820), IFIT3 (*r* = 0.790), IFIT5 (*r* = 0.790) in LAML (all *P*-value < 0.001). The PPI network constructed by STRING database also showed that IFIT family members had a close connection (enrichment *P*-value: < 1.0e-16) (Fig. 2C). Based on GeneMANIA's gene–gene network, IFIT family interacts with 20 potential target genes (Fig. 2D). These results suggested that IFIT1/2/3/5 may function as a complex.

### Survival analysis of IFIT genes in AML

**Fig. 3:**
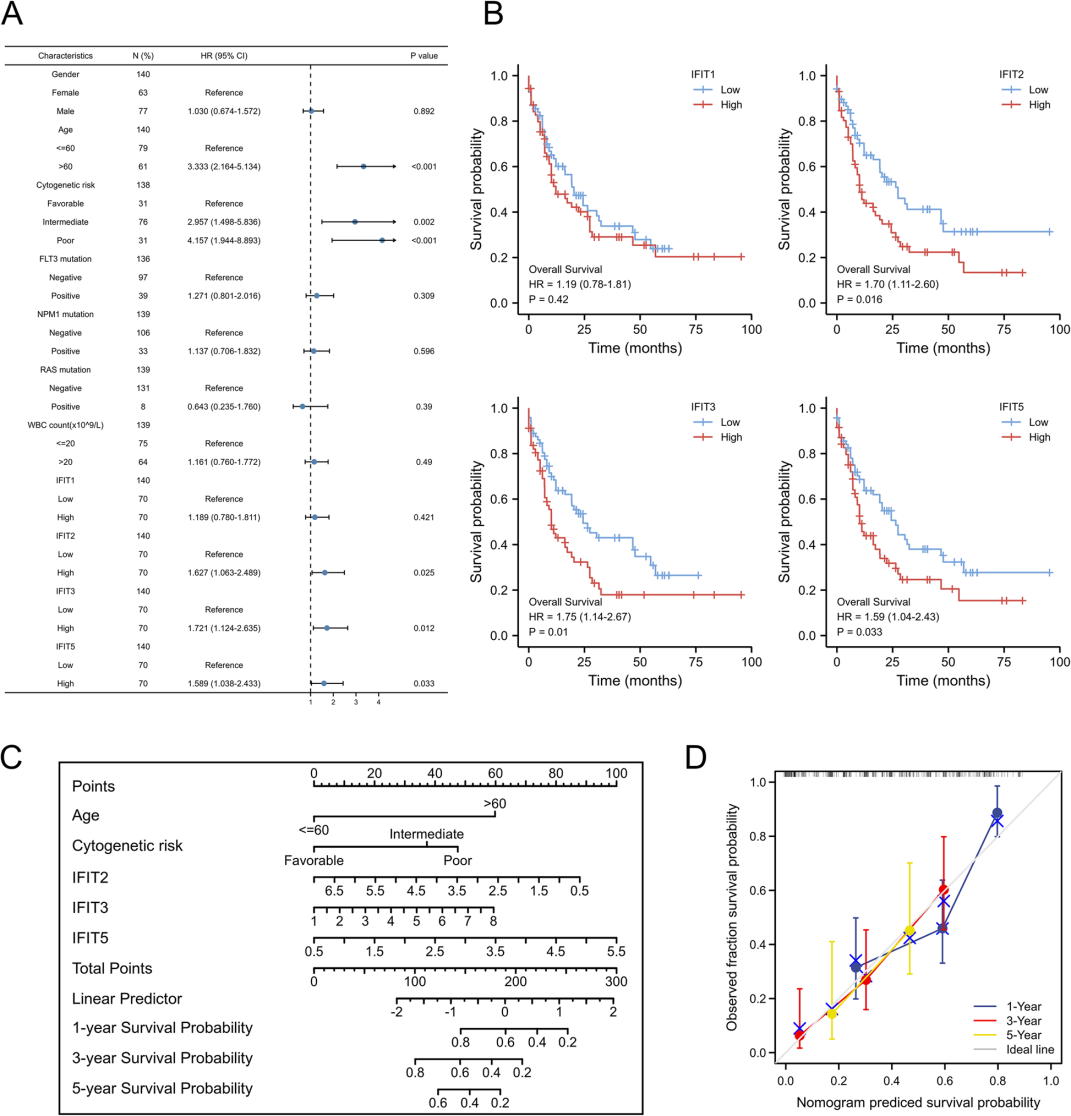
The prognostic value of IFITs in AML. **A** Forest plot for univariate cox regression analysis of IFITs expression for predicting overall survival (OS) in AML with different clinicopathological features. **B** Kaplan–Meier curves for OS of AML patients with high or low expression of IFITs. **C** Construction of nomogram composed of age, cytogenetic risk and the mRNA expression of IFIT2/3/5. **D** The calibration plot for the survival probability at 1, 3 and 5 years of our nomogram

To assess the prognosis significance of IFIT family in AML, we performed the univariate survival analysis which added clinical variates involving patients’ age, gender, white blood cells count, cytogenetic risk, FLT3 mutation, NPM1 mutation, RAS mutation and IFIT mRNA expression level. As a result, age, cytogenetic risk and the expression level of IFIT2, IFIT3 and IFIT5 are associated with the clinical outcomes of AML patients (Fig. 3A). Moreover, the Kaplan–Meier curve also confirmed that AML patients with increased expression of IFIT2/3/5 suffered shorter overall survival compared with patients with lower IFIT2/3/5 expression (Fig. 3B). Given that IFIT2/3/5 expression are potential prognosis predictors, we constructed a nomogram integrating patients’ age, cytogenetic risk and the mRNA expression of IFIT2/3/5 to predict the 1-year, 3-year and 5-year survival probability for AML patients (Fig. 3C). The calibration plot for the survival probability at 1, 3 and 5 years exhibited that the prediction by nomogram and actual observations have a consistent agreement (Fig. 3D).

### DNA methylation analysis of IFIT genes in AML

**Fig. 4:**
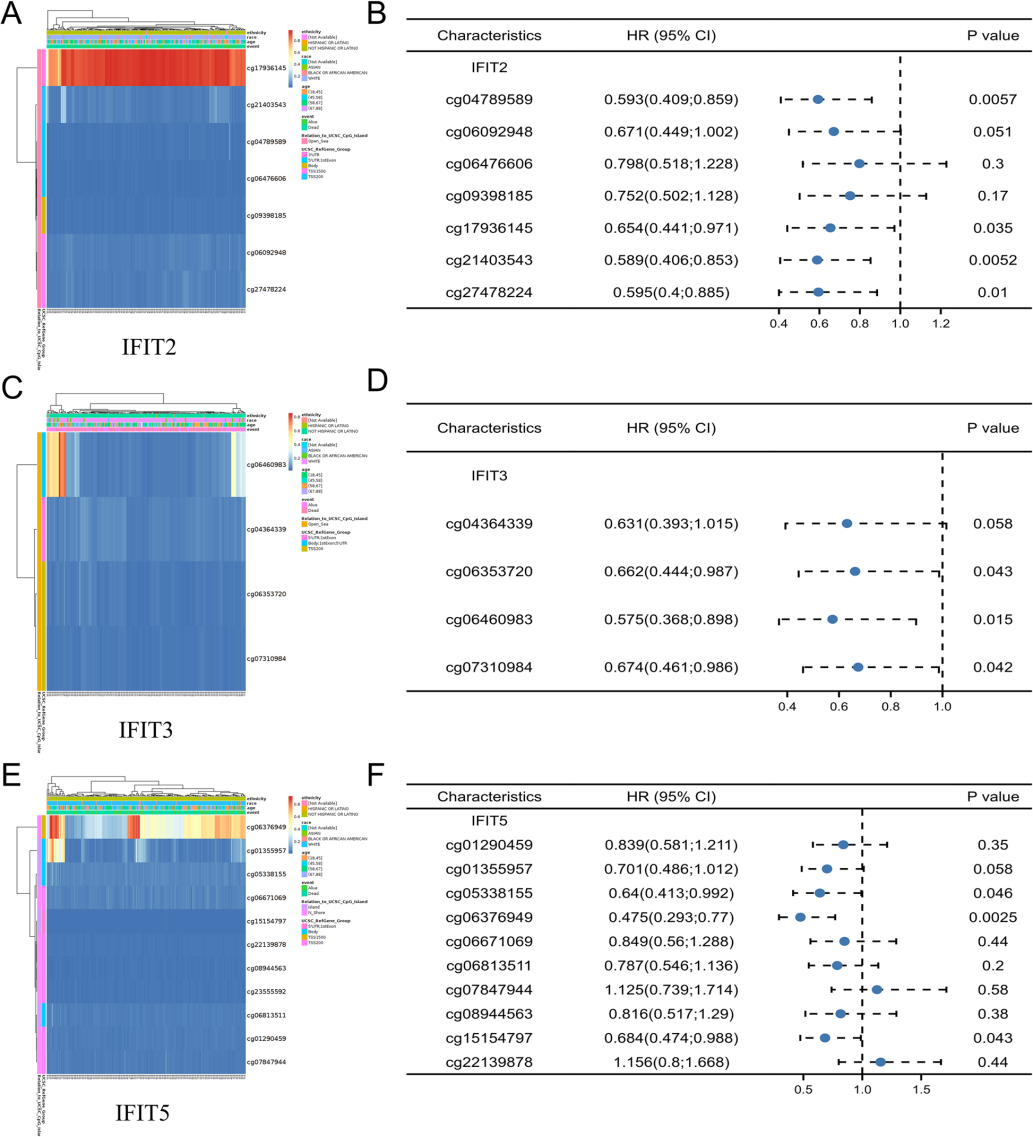
The DNA methylation analysis of IFITs in AML. **A**, **C**, **E** The DNA methylation levels of IFIT2, IFIT3 and IFIT5 in AML respectively. **B**, **D**, **F** The relationship between methylation levels of IFIT2/3/5 and survival of patients with AML

The reason for the dysregulation of IFITs expression in AML is largely unknown. Here we detected the DNA methylation level of IFIT 2, IFIT3 and IFIT5 in AML patients using MethSurv tool. As presented in methylation maps, 7 CpG sites of IFIT2 (Fig. 4A), 4 CpG sites of IFIT3 (Fig. 4C), 10 CpG sites of IFIT5 (Fig. 4E) were found. Among them, low methylation levels of IFIT2 in cg04789589, cg17936145, cg21403543, cg27478224 (Fig. 4B), low methylation levels of IFIT3 in cg06460983, cg07310984 (Fig. 4D), low methylation levels of IFIT5 in cg05338155, cg06376949, cg15154797 (Fig. 4F) were significantly associated with poor prognosis of AML patients. These findings suggested that the pattern of methylation changes of IFIT2/3/5 may be involved in the progress of AML, and were a potential prognosis index.

### Function enrichment analysis of IFIT genes in AML

**Fig. 5:**
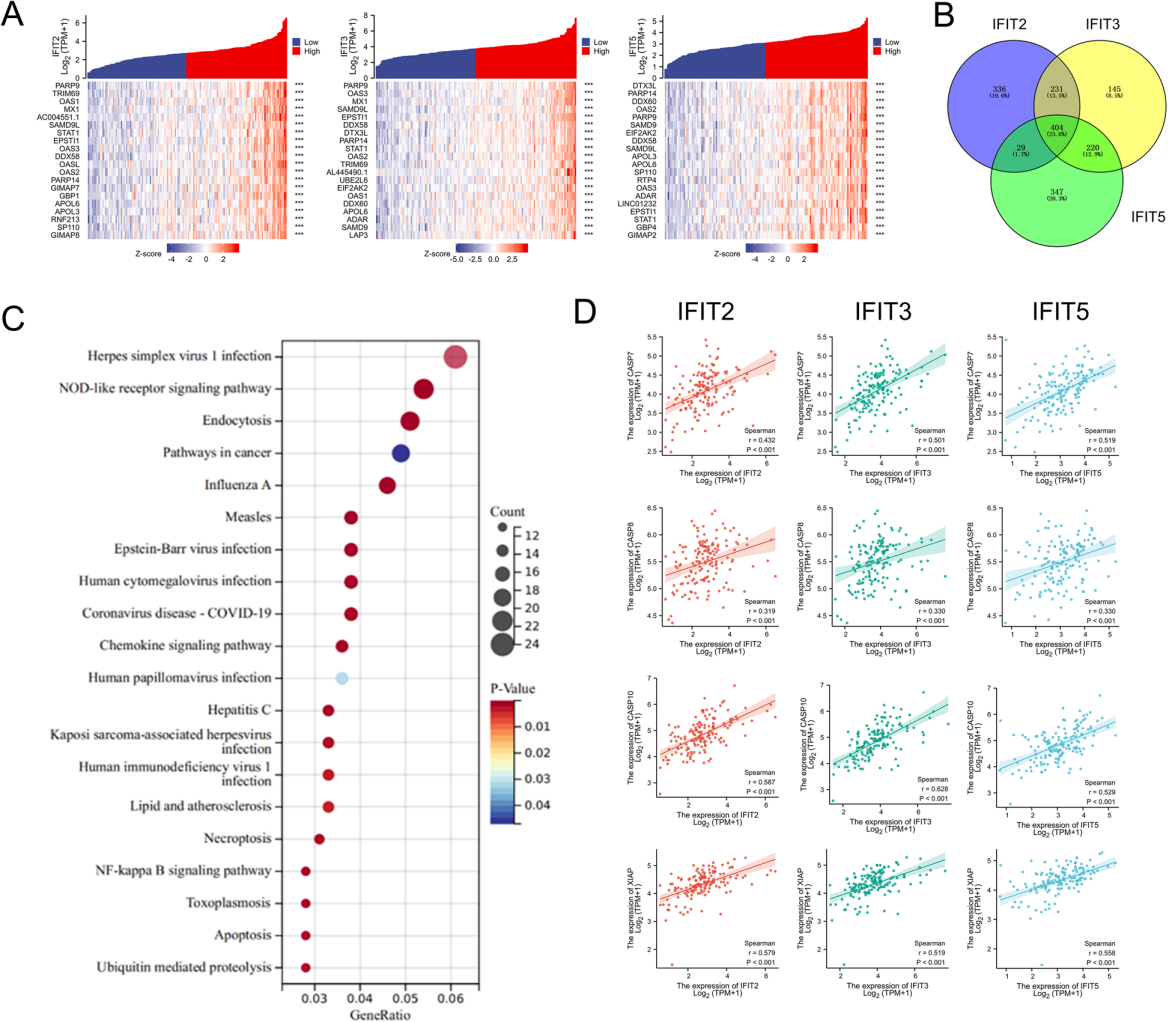
Function enrichment analysis of IFITs-related gene sets in AML. **A **We screened out the first 1000 genes positively related to IFIT2/3/5 based on Spearman correlation analysis. The heat maps exhibited the top 20 genes of IFIT2/3/5, respectively. **B** Identification of the co-correlated genes of IFIT2/3/5 by Venn diagram. **C** KEGG pathways annotations of IFITs and their co-correlated genes. **D** The association between IFIT2/3/5 expression and various apoptosis regulators, including caspase7 (CASP7), caspase8 (CASP7), caspase10 (CASP7) and X-linked inhibitor of apoptosis (XIAP)

Based on the results above, we found that IFIT2/3/5 expression was up-regulated in AML tissues and high expression of IFIT2/3/5 was associated with poor prognosis of AML patients. However, the changes in the signaling pathways caused by increased IFIT2/3/5 expression in AML are as yet unknown. First, we screened the top 1000 genes that were positively related to IFIT2, IFIT3 and IFIT5 respectively, and the heat maps exhibited the top 20 genes (Fig. 5A). By intersecting in the Veen map, 404 co-correlated genes of IFIT2/3/5 were identified (Fig. 5B) (Supplementary Table 1). Function enrichment analysis revealed that these genes enriched in several pathways that involved the pathogenesis in AML, such as “Apoptosis”, “Pathways in cancer”, “Chemokine signaling pathway”, “Necroptosis” (Fig. 5C). Correlation analysis further confirmed that IFITs had a positive correlation with apoptosis regulators, including caspase7 (CASP7), caspase8 (CASP8), caspase10 (CASP10) and X-linked inhibitor of apoptosis (XIAP) (Fig. 5D).

### Immune infiltration analysis of IFIT genes in AML

**Fig. 6:**
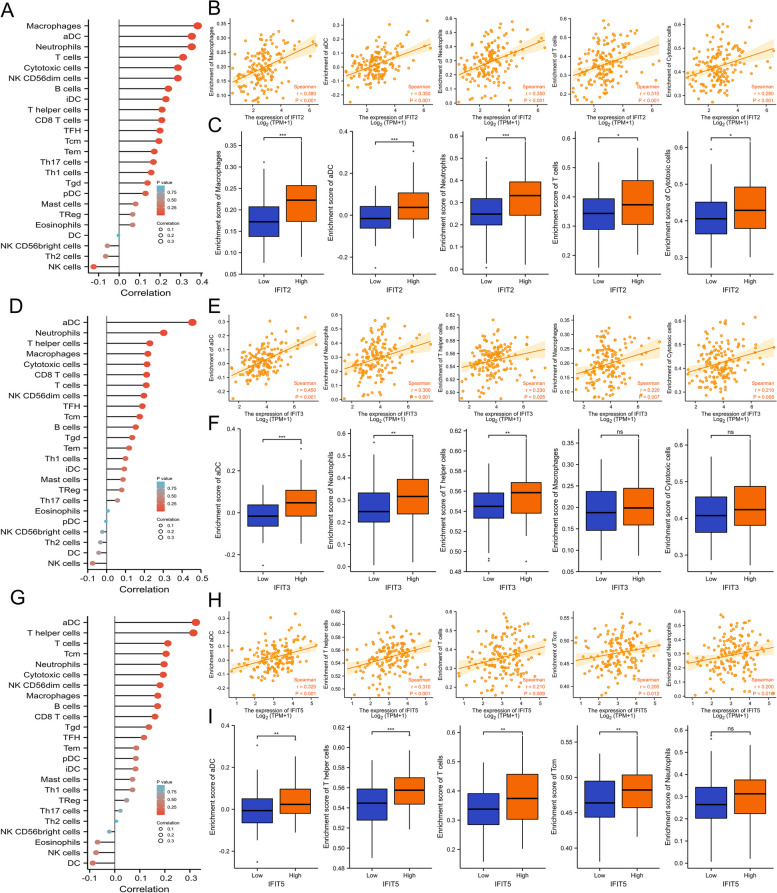
Spearman’s correlation of IFIT 2/3/5 expression with 24 types of the immune cells’ infiltration levels. **A**, **D**, **G** The forest plot shows the relations between the abundance of 24 immune cells and IFIT 2/3/5 expression. **B**, **E**, **H** The scatter diagrams of the top 5 immune cells displayed the greatest correlation with IFIT 2/3/5 expression. **C**, **F**, **I** The level of the top positive-related 5 immune cells was higher in IFIT 2/3/5 high expression group compared with the low expression group. *P < 0.05, * *P < 0.01, ***P < 0.001

Next, we evaluated the correlation among IFIT2/3/5 mRNA expression and the infiltration levels of 24 types of immune cells. As the lollipop diagrams presented, IFIT2/3/5 genes were significantly associated with multiple immune cells. Specifically, macrophages, activated Dendritic Cells (aDC), neutrophils, T cells and cytotoxic cells were the top five immune cells with the highest correlation with IFIT2. In addition, the infiltration levels of these immune cells were significantly higher in IFIT2 high-expression groups compared with IFIT2 low-expression groups (Fig. 6A-C). Similar findings were observed for IFIT3 and IFIT5 (Fig. 6D-I). These data implied that the IFIT family may play a critical immune-regulation role in AML by influencing immune cell infiltrates.

### Correlation analysis between IFIT genes and immune checkpoints in AML

**Fig. 7:**
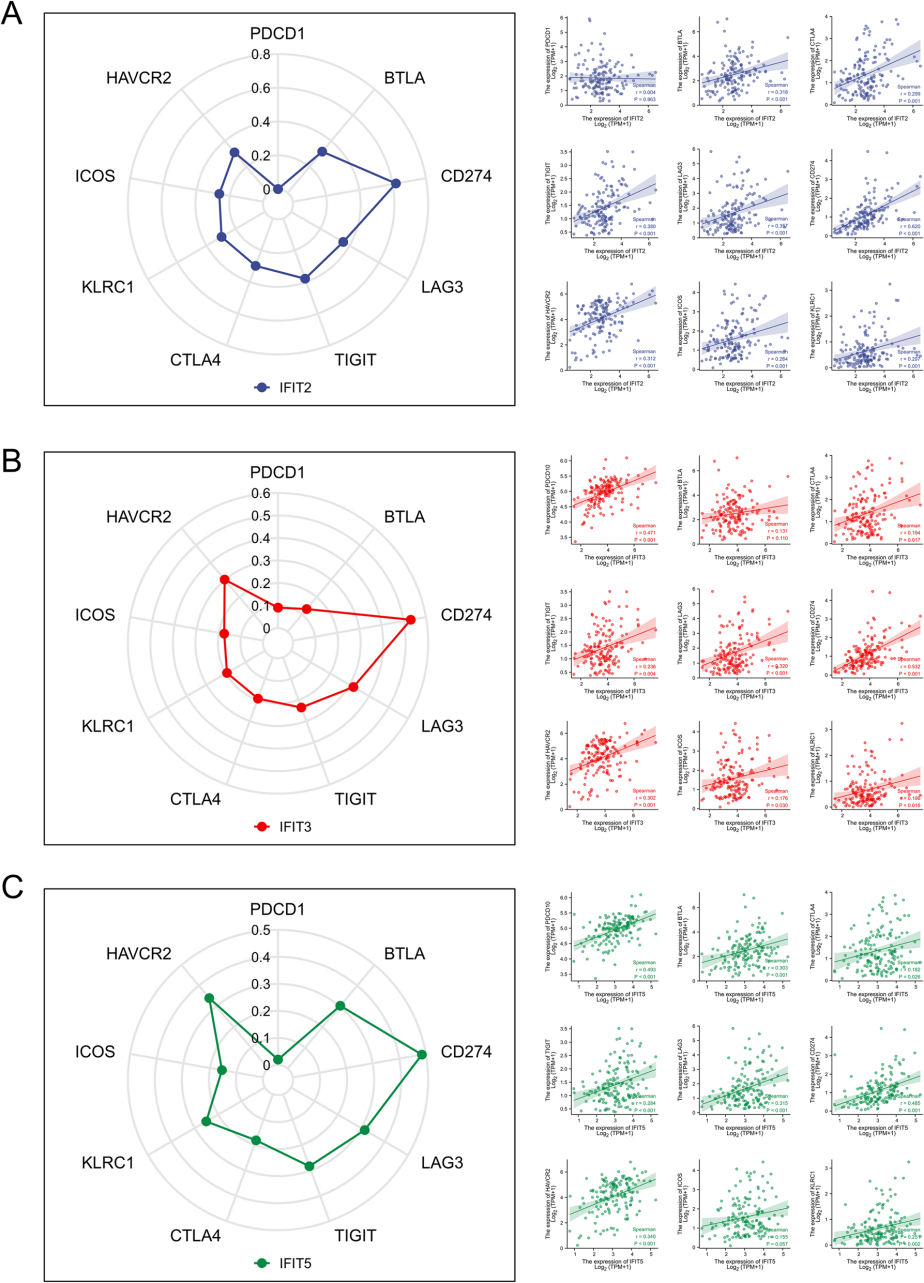
Relationship between IFITs expression and immune checkpoints. **A**, **B**, **C** The radar plots and scatter plots exhibit the positive correlation between IFIT 2/3/5 expression and immune checkpoints, including PDCD1, BTLA, CTLA4, CD274 (PD-L1), LAG3, TIGIT, KLRC1, ICOS and HAVCR2

Considering the important role of immune checkpoint molecules in tumor immune response, the relationships between IFIT2/3/5 mRNA levels and immune checkpoints expression in AML were also explored through TCGA database. Several famous immune checkpoints are included in our analysis, such as PDCD1 (PD1), BTLA, CTLA4, CD274 (PD-L1), LAG3, TIGIT, KLRC1, ICOS and HAVCR2 (TIM-3).As a result, IFIT2 had a significant positive correlation with BTLA (*r* = 0.318), CTLA4 (*r* = 0.299), CD274 (*r* = 0.620,), LAG3 (*r* = 0.357), TIGIT (*r* = 0.380), KLRC1 (*r* = 0.297), ICOS (*r* = 0.264) and HAVCR2 (*r* = 0.312) (all *P*-value < 0.001) (Fig. 7A). IFIT3 had a significant positive correlation with PDCD1 (*r* = 0.471, *p* < 0.001), CTLA4 (*r* = 0.194, *p* = 0.017), CD274 (*r* = 0.532, *p* < 0.001), LAG3 (*r* = 0.320, *p* < 0.001), TIGIT (*r* = 0.236, *p* = 0.004), KLRC1(*r* = 0.196, *p* = 0.016), ICOS (*r* = 0.176, *p* = 0.030) and HAVCR2 (*r* = 0.302, *p* < 0.001) (Fig. 7B). Meanwhile, IFIT5 had a significant positive correlation with PDCD1 (*r* = 0.493, *p* < 0.001), BTLA (*r* = 0.303, *p* < 0.001), CTLA4 (*r* = 0.182, *p *= 0.026), CD274 (*r* = 0.485, *p* < 0.001), LAG3 (*r* = 0.315, *p* < 0.001), TIGIT (*r* = 0.284, *p* < 0.001), KLRC1 (*r* = 0.251, *p* = 0.002) and HAVCR2 (*r* = 0.340, *p* < 0.001) (Fig. 7C).

### Drug sensitivity analysis of IFIT genes in AML

**Fig. 8:**
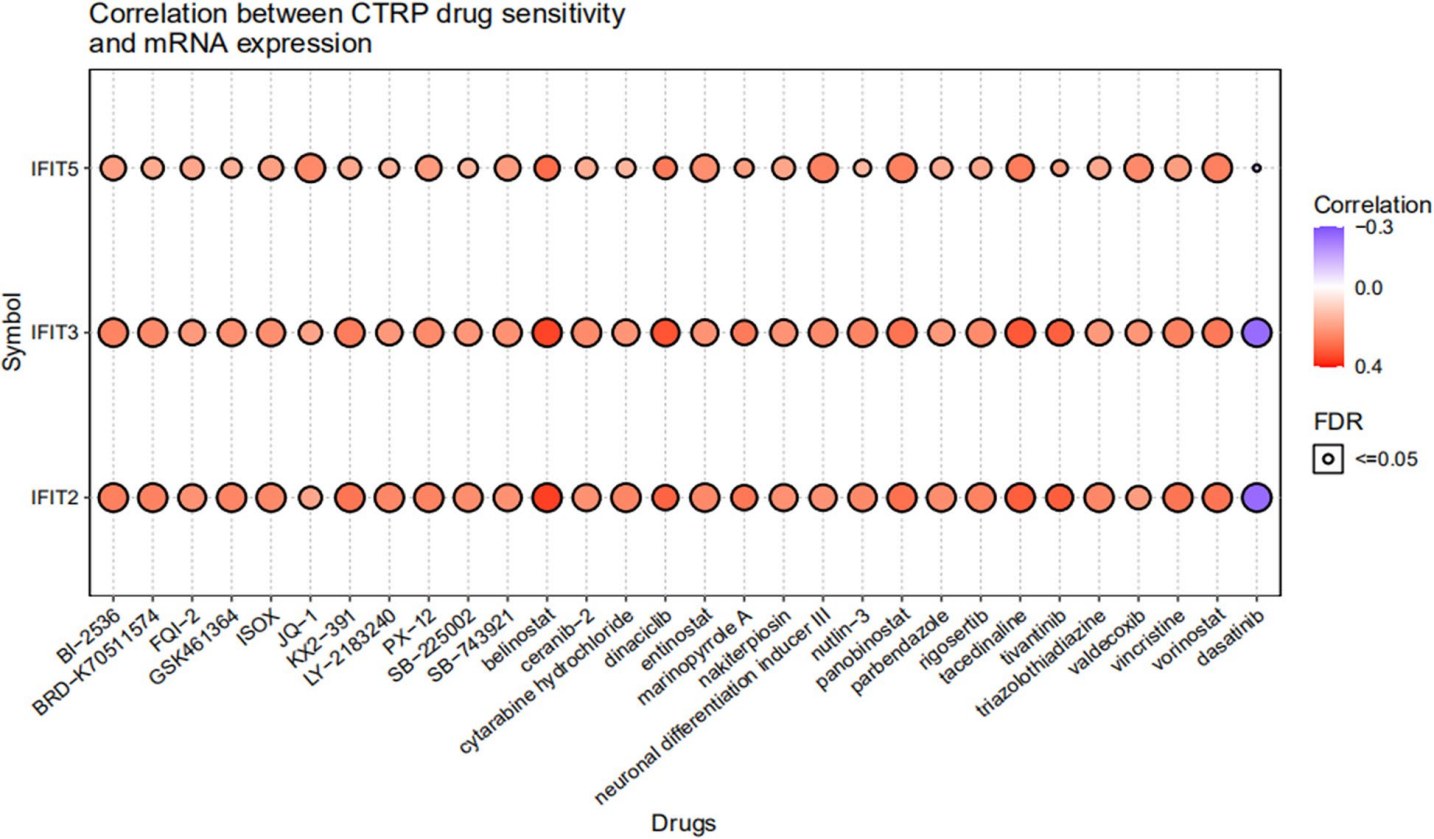
Drug sensitivity analysis of IFIT family. The heatmap showed the correlation between IFIT2/3/5 mRNA expression and biochemical half maximal inhibitory concentration (IC50) data of various drugs or small molecules based on the CTRP database. The positive correlation means that the gene high expression is resistant to the drug, vise verse

Studies in the past have shown that the IFIT family was involved in anticancer drug resistance [24]. Therefore, we evaluated the relationship between IFIT2/3/5 mRNA expression and biochemical half maximal inhibitory concentration (IC50) data of 481 drugs or small molecules based on the CTRP database. As a result, the expression of IFIT2/3/5 was positively correlated with IC50 of multiple drugs, such as cytarabine hydrochloride, entinostat and belinostat, but negatively correlated with the IC50 of dasatinib (Fig. 8). These findings suggested that AML patients with high expression of IFITs were resistant to many drugs, but may be sensitive to dasatinib.

## Discussions

Among hundreds of IFN-stimulated genes, IFIT gene family are most highly responsive ISGs and regulated by the JAK-STAT signaling pathway [25]. In the past few decades, IFIT family members have been well studied in the antiviral immune response [26]. In contrast to the relatively unambiguous antiviral role, the functions of the IFIT family in the pathogenesis of different tumors have not been defined clearly. Recent structural and functional studies have implicated that IFITs are involved in tumor initiation, progression and treatment resistance [25]. However, the prognosis role and biological significance of IFIT family members in acute myeloid leukemia has not yet been elucidated. To our knowledge, this is the first comprehensive bioinformatics analysis of the IFIT gene family in AML.

In the present study, we first focus on the expression level and prognostic role of IFIT family in AML. Consistent with past studies, the expression of IFIT1/2/3/5 was dysregulated in multiple types of human tumors, including bladder cancer, pancreatic cancer, oral squamous cell carcinoma and neck squamous cell carcinoma [26]. In AML, the four genes included in IFIT family were significantly up-regulated, with a high positive correlation among four members (r > 0.6, *p* < 0.001). Previous multiple studies have identified IFIT members as potential biomarkers for tumor diagnosis, prognosis and treatment response. As an example, high expression of IFIT3 predict better response to IFN-α therapy in patients with hepatocellular carcinoma [27]. Jiang et al. revealed that IFIT1/2/3/5 genes could serve as diagnosis markers for skin cutaneous melanoma (SKCM), and predicted the survive outcome of SKCM patients [28]. In our analysis, the ROC curve showed that IFIT1/2/3/5 had a high ability to distinguish AML from normal blood tissues (AUC > 0.9), suggesting that IFIT family members are promising diagnostic biomarkers for AML. In addition, regression analysis revealed that the high expression of IFIT2/3/5 was associated with the poor prognosis of patients with AML. The Kaplan–Meier curve also confirmed that AML patients with high expression of IFIT2/3/5 suffered shorter overall survival compared with low expression groups. Taken together, IFIT family genes might serve as diagnosis and prognosis biomarkers for AML. Consistent with past studies [29, 30], our regression analysis also showed that older age (> 60) and high cytogenetic risk were independent factors predicting unfavorable prognosis of AML. Therefore, a nomogram prognosis model combining IIFIT2/3/5 expression with age and cytogenetic risk was developed to obtain a more accurate prognosis prediction model. As seen from this perspective, our model may be able to provide an individual score for patients with AML.

Next, we evaluated the changes of signaling pathway caused by increased IFIT2/3/5 expression in AML. A total of 404 co-correlated genes of IFIT2/3/5 were identified, and KEGG analysis indicated that these genes associated with several pathways, such as “Pathways in cancer”, “Necroptosis” and “Apoptosis”. Furthermore, correlation analysis showed that IFITs was positively correlated with apoptosis regulators, including CASP7, CASP8, CASP10 and XIAP. Indeed, previous studies have shown that IFIT-family proteins play important roles in cell apoptosis. In this case, IFIT2 ectopic expression was found to induce the activation of caspase-3, a key mediator of apoptosis, and disturbed the asymmetry of the plasma membrane [31]. Feng et al. demonstrated that miR-645 altered the caspase-3/7 activity and inhibited the cell apoptosis of gastric cancer by down-regulating the expression of IFIT2 [32]. Regarding to IFIT3, IFN-α-induced IFIT3 expression led to the activation of apoptotic regulators, including caspase 3/8/9 and Bcl-2-associated X protein [33]. These results indicated that IFITs overexpression might affect the tumorigenesis and progression of AML through apoptosis pathway.

There are growing evidence that bone immune microenvironment plays an important role in immunotherapy, tumor progression and influences the prognosis of AML patients [33, 34]. Therefore, we next evaluated the relationship between IFITs expression and immune cell infiltration in AML microenvironment. We found that IFIT2/3/5 was positively correlated the abundance of several immune cell, such as macrophages and T-helper cells. A recent study found that leukemia-associated macrophages protect AML cells from apoptosis and are related to chemotherapy resistance [35]. T-helper cells have been implicated in the development of AML in previous studies [36–38]. These results suggested that IFITs might influenced the prognosis of AML patients through modulating immune microenvironment.

It has been confirmed that tumor microenvironment contains a high number of immunosuppressive cells and molecules, which can up-regulate the expression of immune checkpoint receptors in T cells, resulting in the loss of anti-tumor role of T cells and triggering immune escape of tumor cells [39, 40]. Due to this, we further assessed the correlation of IFITs expression and several famous immune checkpoints in AML. As a result, IFIT2/3/5 had a significantly positive correlation with the expression of these immune checkpoints, such as PD1, PDL-1, CTLA-4 and LAGs. Indeed, a past study confirmed that IFIT2 is significantly up-expressed in patients who are not sensitive to anti-CTLA-4 therapy in metastatic melanoma [41], suggesting that the IFIT family may be useful for predicting the efficacy of immune checkpoint therapy. Taken together, IFIT family genes may be important immunoregulator for AML.

Despite advances in treatment, AML remains a challenging disease due to drug resistance and disease relapse [42]. Recently, IFIT family members has been implicated in anticancer drug resistance [43]. For example, overexpression of IFIT1 or IFIT3 increased oral squamous cell carcinoma (OSCC) resistance to multiple chemotherapeutic drugs, including 5FU, cisplatin, oxaliplatin, carboplatin [24, 44]. On the contrary, the high expression of IFIT1 and IFIT3 in OSCC contributes to gefitinib's anti-tumor effect by enhancing p-EGFR recycling [45]. In our analysis, drug sensitivity test results showed that high expression of IFITs in AML was resistant to various drugs or small molecule, but sensitive to dasatinib. As a famous tyrosine kinase inhibitor, dasatinib was widely used in chronic myeloid leukemia and Philadelphia chromosome-positive acute lymphatic leukemia. Therefore, our study might provide an additional selection for drug-targeted therapy of AML, and IFIT family members might be useful markers for monitoring drug resistance.

In summary, all four IFIT family members were overexpressed in AML patients, and had ability to discriminate AML from normal tissues. Survival analysis revealed that AML patients with high expression of IFIT2/3/5 had a poor prognosis. Besides, IFIT2/3/5 expression was significantly positively correlated with immune cell infiltration and immune checkpoint expression. In addition, drug sensitivity analysis showed that AML patients with upregulation of IFIT2/3/5 were resistant to various drugs but sensitive to dasatinib. In summary, IFIT family members might be biomarkers for diagnostic, prognosis and drug resistance, and promising therapeutic targets for AML patients.

### Supplementary Information

**Additional file 1:** **Supplementary Table 1.** The top 1000 genes positively correlated to IFIT2/3/5 and 404 co-correlated genes of IFIT2/3/5 by Spearman correlation analysis.

## Data Availability

The datasets provided for this study can be found and accessed in online databases. These online databases were accessible from the following addresses. TCGA official website (https://www.cancer.gov/tcga). (GTEx) database (http://www.gtexportal.org). GSCA database (http://bioinfo.life.hust.edu.cn/GSCA/). GEPIA database (http://gepia.cancer-pku.cn/index.html). STRING database(https://cn.string-db.org/). GeneMANIA database(http://genemania.org/). MethSurv database (https://biit.cs.ut.ee/methsurv/).
