# Supplementary material for: The diagnostic/prognostic roles and biological function of the IFIT family members in acute myeloid leukemia
Source: BMC Med Genomics. 2023 Nov 18;16:296. doi: 10.1186/s12920-023-01735-0 (PMC10657597; doi:10.1186/s12920-023-01735-0)
Supplement: Supplementary file 1 — Additional file 1: Supplementary Table 1. The top 1000 genes positively correlated to IFIT2/3/5 and 404 co-correlated genes of IFIT2/3/5 by Spearman correlation analysis. [file 12920_2023_1735_MOESM1_ESM.docx]

Supplementary table1. The top 1000 genes positively correlated to IFIT2/3/5 and 404 co-correlated genes of IFIT2/3/5 by Spearman correlation analysis.

| **Correlated Gene of IFIT2** | **Correlated Gene of IFIT3** | **Correlated Gene of IFIT5** | **Co-correlated genes of IFIT2/3/5** |
| --- | --- | --- | --- |
| PARP9 TRIM69 OAS1 IFIH1 MX1 AC004551.1 SAMD9L STAT1 EPSTI1 IFIT5 OAS3 DDX58 OASL OAS2 PARP14 GIMAP7 GBP1 IFI44 APOL6 APOL3 RNF213 SP110 GIMAP8 DTX3L AP001610.2 SAMD9 GIMAP6 IRF2 TRAFD1 SIGLEC1 LAP3 GIMAP4 IFI44L SOD2 STAT2 GIMAP2 TLR1 PARP12 RAB8B DDX60 ZDHHC4P1 AL445490.1 AKAP13 APOL2 SGMS1 GIMAP1 ABCC3 ADAR PTPRC FCGR3A NBR1 GIMAP5 CASP5 SAMD4A BATF2 N4BP1 AC009950.1 ZMYND15 IRF9 PHF11 CXCL16 CASP10 Z95114.3 GBP2 CEMIP2 APOL1 TUT7 HPSE SLC9A9 BTN3A3 TAP1 CLEC7A CXCL10 AC124319.3 EIF2AK2 HELZ2 PLSCR1 KLF3 CLEC1A IFI16 SNTB2 BACH1 RN7SL105P ZFP36L1 RNF213-AS1 CCR5 HLA-E CREBL2 GBP3 UBE2L6 OSBPL11 JAK2 CHST15 CTSS JAK1 CMKLR1 IL10RA AL683842.1 SETX RNF149 LIPA CLCN3 SECTM1 GBP5 ST3GAL5-AS1 TMCC3 TNFRSF8 HFE SERPINB9 RIPOR2 RUBCN SIPA1L1 CARD16 SGTB AL031985.1 PECAM1 ADD3 SEC14L1 IRAG1 IFNAR1 SNX6 AC124319.2 LYN DDX60L IL16 TCP11L2 TGFBR2 NRBF2 NCOA3 RASSF3 AC010247.1 OGFRL1 WIPF1 CD274 STX12 ZNF143 LY96 HMOX1 LINC02432 ARHGEF3 CORO1C JAML TLR7 SLC7A7 IQSEC1 KIAA0513 AP3S2 TAB2 SP100 GPR65 ANKRD13A ATP6V1A RASSF5 ETV7 MSR1 ST3GAL5 SRC AC245884.11 SLFN5 TTYH2 SERINC1 MAP3K3 GPR155 LRRK2 APOBEC3G MGC16275 GNGT2 ZNFX1 MAML2 PXN RNF144B PSMB9 KSR1 WBP1L TNFSF8 BCL2A1 NECAP2 SCARB2 CXCL11 BIN2 PICALM SNAP23 GBP4 HERC6 IL15 MORC3 AC106712.1 AL356056.2 AC124319.1 TRIP4 ABI3 ALDH1A2 RESF1 BIRC3 STK4 PLEKHO2 RPL7AP64 CHMP5 LINC01547 LTA4H KDM1B PIP4K2A PDCD1LG2 MARCHF1 RNF141 TLR6 XIAP KLHL5 RSAD2 SLC18B1 ZNF217 PLCL2 MARF1 NUMB ITSN2 RNF13 STAT3 CD300E FGD6 CCDC69 KMO AIDA PATL1 LILRB1 HCP5 XAF1 DPYD AC007000.4 CASP1 AL844908.1 CASP4 CERT1 SLC37A3 SLC43A2 IL21R MYD88 EPHB2 FAM120AOS LIMS1 AC069547.1 CRTAM VNN2 LIPN DRAM2 LILRA5 TNFSF10 SUSD6 EFCAB14 CCR5AS AC113404.3 ARHGAP18 HERC3 KDM7A AC004466.2 MPZL3 MAPK1 AP002954.1 SYNJ1 KAT2B TMEM140 IGSF6 LMBRD1 MX2 LPGAT1 PTP4A2 CLEC4F MIR4632 CCRL2 FAM118B KIAA0040 SLC16A6 GPRIN3 UBE2F RGS2 RTP4 PSMB8-AS1 BTN3A1 GLTP STK38 ZFAND3 TAP2 LINC02528 PDPK1 SLC31A1 CMPK2 FBXO8 PCYT1A CNEP1R1 AC245884.2 CTDSP2 HSPE1P18 RABGAP1L ZBTB38 SBF2 TMEM127 ASB7 RAP1A KIDINS220 SH3TC1 CARD6 RNF111 IL6ST HECA LILRB2 VIPR1-AS1 MOB3C IL27RA CAB39 PHTF2 IFIT3 IFIT1 PPP2R5C ZNF106 RPS20P31 MTMR12 AC245884.12 CD300A RPS12P20 AC034199.1 CCNY LINC02185 BICRAL FOLR2 ITM2B RGL1 TCF7L2 FNIP2 RIC1 YBX2P1 XKR7 GBP1P1 STX7 GLG1 PIAS1 GVINP1 RAPH1 DAPP1 SLC49A4 CEP85L LINC01232 ATP7A RHOC NOTCH2 NBPF10 KCTD12 CLIP1 AL121985.1 FGL2 NLRP1 AL136295.5 SRD5A1 C10orf105 RETREG3 TLN1 TNFRSF1B NAGK ADGRE3 YIPF1 WASF2 AC099343.2 LINC02085 C5AR1 PSAP CLIP4 SLAMF8 ST6GAL1 CDH23 AQP9 USP18 GNA13 HEG1 MIR548AT AL032821.1 TICAM2 TMEM268 UHMK1 MR1 LILRA1 RCBTB2 MEFV SULF2 KLHDC7B-DT NUAK2 RAB9A RPGRIP1 RAP1B GRK3 CYTIP TLR8 LINC01094 AL353751.1 MTF1 MIR4718 CX3CR1 PSEN1 HCFC2 WARS1 ADAM10 PPP2R5A CARD14 VIPR1 LINC01881 U3 SLC12A6 ARHGAP30 MIR6075 AC023825.1 FKBP15 RECQL CD48 FMR1 AC109597.1 PDK4 AC116348.3 ABHD2 ZNF710 AC087286.4 WASHC4 CPNE3 UBR2 RAB10 MFSD11 HSPA6 MTND4P23 BROX AL512444.1 MTSS1 KLHL6 CDC40 AC116348.2 AC067852.3 CAMK1D FFAR2 NCOA2 FYN CD46 VMO1 DHX58 MICAL2 LRP1 EXOC1 ARAP1-AS2 MAF AC026470.2 ARAP1 WNK1 SYNRG CYTH1 CDC73 MYOF SDC3 SLC26A11 GPAT3 AC003070.1 IRAG1-AS1 PRKCB MIR4518 RAB29 TMEM131L RAB3GAP1 VCL ZNF845 AC007919.1 DOCK8 AC015845.1 CEPT1 LINC00877 Y_RNA GNAI3 NCK1 SIGLEC11 FAM13A P2RX7 TMCC1 MEF2C PPP1R27 TMBIM1 MICU1 BIN2P1 INPP4A MBD2 AC109597.2 SLC37A2 LATS1 ZDHHC20 SAMD8 DOCK11 RAB22A AL020997.3 STK40 SH2B3 CCNG1P1 GOLGA5P1 BTBD10 SNORD56B ZSWIM6 SLA WDR44 AC012511.1 ERBIN KCNH3 TRIM25 USP25 ARHGAP27 FCGR2C LINC01146 SPG11 ACSL4 IFI35 TNKS2 PIK3AP1 LCP2 SLC15A3 SH3GLB1 NPC1 STX6 ADAP2 RAB11A AC004921.1 SEMA4D IGF2BP3 IFI30 MSNP1 GDPD1 UTRN MTCO3P23 HLA-F LAMTOR3 MSL3 ITGB1 PTPRO CDC42EP2 RABGAP1 TATDN3 PLD2 C9orf72 IFIT6P LRP1-AS OSBPL8 AMZ2P2 PA2G4P6 LACC1 CHST7 CHURC1 C9orf106 TRIM34 SIDT2 NBPF14 NIN MTND5P32 CXCR2P1 GIN1 FPR3 LPIN2 NCOA1 CDS2 RAB11FIP4 TOMM20P2 ZNF267 FGD3 LYPD2 CLEC4A CARMIL1 AFTPH USP3 CPPED1 ISG15 RN7SL683P SPATA13 SECISBP2L AC099552.2 PDP1 AC083862.1 PDK3 SCIMP MAP3K14 RC3H2 TESK2 KIAA0232 RNF20 GPT2 NFE2L2 GPBAR1 PCDH12 RNF145 RNU7-61P MBNL2 AC245036.3 FAM32BP VTI1B R3HCC1L SYNE1 CCM2 SNX18 VPS26C PRKACA RBM18 CREBRF BIRC2 HELZ AC078845.1 ARRB1 AC116348.1 IQGAP2 RDH11 ZNF467 UBE4A TNFSF15 ZNF699 RAB39A SIGLEC16 VAV1 SNX29 FPR2 DICER1 NSL1 NUPR1 AIM2 ITCH AC118344.2 CDC42P3 ACOX1 TXNIP LILRA6 CLCC1 BTG2 CREB5 NUB1 TINF2 LRRC8D AL137186.2 CTSO USP15 CRK SLC25A10 AL589935.1 AC092145.1 WAC APOBEC3A HOOK3 OSTM1 ZDHHC7 MIR4323 NMI MSN AC111170.4 ABI1 VNN3 MAP3K5 LILRP2 TMEM132A SLC4A8 MAP2K1 TMEM131 ANKRD44 CAP1 SNAPC3 SGK1 SLC46A2 PRKCH EXOC8 CREBBP ARL6IP5 AC100803.2 LINC01948 CRTC3 KCTD7 FGD2 CD300LD SPTLC1 C6orf62 C6orf120 EXT1 MYCL HCCS APC AC005476.2 ACOT9 MAT2B TMPRSS13 SNAP29 SSH2 KIF13A TMEM167A ATF6 STOM DHX57 SP3 TCEANC KDM5A ARHGAP25 PKD2L1 ITGAL ACVR1 GPCPD1 CD52 NFKBIZ LINC02773 UBR3 CHMP2B ZNF436 SNX11 TGFBI SSH1 BTN3A2 B2M RNU6-859P UBQLN2 SLC2A6 FPR1 MAFB KATNBL1P5 ELK3 VAPB RPS6KA3 Y_RNA ALKBH1 SASH1 PRAL AP003733.3 IFITM3 AC066616.2 TOX4 SLAIN2 C1GALT1 GALNT10 FOXN3-AS1 C6orf89 TAGAP NEDD9 ANXA4 PPFIA1 RAP2B FAM20A MTCYBP23 STYX SWT1 ZNF24 ZNF703 TSC22D2 PAPOLG BCL10 C17orf77 AC087741.1 PHOSPHO1 CDC42SE1 LILRB3 ETV7-AS1 SLC6A6 CTTNBP2NL POU2F2 EPS8 UBE2Q2P9 NEURL3 AC105265.2 IL1B AF124730.1 VN1R105P DENND2D AGPAT3 MMAA AC021739.3 PANX1 CYLD P2RY13 EHBP1L1 SLC30A1 BNIP3L TMEM229B CRLF3 AL157871.3 DAPK1 RPS10P5 GSKIP SLC44A2 CASP7 AC126615.2 SLAMF7 HMGB1P3 CLEC10A NLRP12 HORMAD1 AC078962.3 NOTCH2NLA AL354813.1 HERC1 AC103739.1 FTH1P16 XRN1 DYNC1LI1 IFNGR1 LINC02384 CCDC137P CD14 AC025040.2 ARAP2 KCTD21 MOB1A NLRC4 MARCO PAOX CAPZA2 RAB12 CCDC93 UBL3 C1QA PPM1M GOLGA5 GDAP2 GUCY1B1 AL391314.1 TM2D2 RPS2P15 TLR5 WDR11 SEPTIN9 FOXN2 ERCC4 SP1 TAGLN PTGS2 FLVCR2 KIF13B STAM2 CTR9 HDAC7 TEP1 TAB3 TRIM22 SLC8A1 FCGR3B SDCBP Y_RNA MTPN KDM7A-DT MALT1 VPS13D CARD17 ATP6V0A1 KCTD20 AC108206.1 AL024509.1 ACAP2 TBC1D23 PIK3R5 PAPSS2 LYSMD2 RXRA SLC25A30 UST BMP2K EML4 AC069528.2 LILRB5 AL356481.1 USP38 TRIM23 DDI2 PLAAT4 AC064805.1 NHEJ1 DAB2 SEC63P1 ITM2BP1 HK1 RBBP5 RPS6KC1 FBXL3 FTH1 SFT2D2 ARRDC3 SLC6A12 MFAP3 VCPIP1 AL023807.1 AC004241.5 STK24 GPX3 LUCAT1 MIR3174 BATF3 ARMH3 ELOA NMT1 PEA15 ITPR1 AC010654.1 BRAP TMLHE CD200R1 SIGLEC9 SLC9A7 AC138207.1 CD86 JKAMP FTH1P23 RAB5A TTC39B NEK7 STIM2 CMTR1 OTULIN LINC01503 CPVL-AS1 MIR29B2CHG AC048337.1 HEIH CD40 LINC00299 AC004241.1 AC144831.1 LFNG GIHCG EPS15 IL15RA RYBP MTHFR RPS12P26 RHOT1 SETBP1-DT NAGS ZNF821 TCF20 FAM126A PARP11 LMTK2 MTMR1 FGD4 POLK PDCD10 H3C6 TRAF3IP3 AC104447.1 MTMR10 GLCE PIK3C2A FTH1P10 TRMT1L MAP3K7CL MT-CO2 FUCA1 AC078843.1 SPOP AC008033.3 IRF7 P2RY12 FOXN3 AC073850.1 AC130454.1 GCNT2 RNASEL SNX21 AC020633.1 AC004466.3 CUL4B DAPK1-IT1 HPS5 RN7SL172P HSPA7 MTND3P12 RPL21P136 ECE1 CD36 AL512506.3 BMF CGRRF1 IGF2R DR1 USP33 FMNL2 BLVRA CCR2 BACH1-IT1 EP300 RASA3 AC105265.1 PDCD6IP OXSR1 YBX1P8 IKZF1 GCH1 PLPPR3 BECN1 LINC02757 FEM1B TNK2-AS1 NAMPT MYOM1 GLIPR2 ZNF702P FOXO1 WDR26 NLRC5 MARCHF7 CCDC88C GOSR2 MIR7848 CLTC OLFM1 AC022217.3 | PARP9 OAS3 IFIH1 MX1 SAMD9L IFI44 EPSTI1 DDX58 DTX3L IFIT5 IFI44L PARP14 STAT1 OAS2 TRIM69 AL445490.1 UBE2L6 EIF2AK2 OAS1 DDX60 APOL6 ADAR AC004551.1 AP001610.2 SAMD9 LAP3 OASL RSAD2 APOL3 SP110 GIMAP6 RNF213 HERC6 GBP1 APOL1 GIMAP2 CASP10 CMPK2 N4BP1 APOL2 IRF2 PARP12 SOD2 IFI16 GBP1P1 SIGLEC1 RTP4 LIMS1 IRF9 ZDHHC4P1 GBP3 USP18 CHMP5 BTN3A3 PHF11 GIMAP7 NBR1 GBP4 AC009950.1 Z95114.3 HELZ2 SGMS1 JAK1 APOBEC3G CXCL10 PLSCR1 GIMAP8 DHX58 TAP1 RAB8B HLA-E PSMB9 STAT2 TRAFD1 BATF2 ETV7 AL353751.1 PECAM1 LIPA CLCN3 WIPF1 IFI6 RBM18 PIP4K2A NMI SLC12A6 XAF1 MORC3 SNX6 SP100 ASB7 PTPRC CD274 DDX60L WBP1L ZFAND3 SEC14L1 CMTR1 ANKRD13A RAB3GAP1 USP38 STAT3 AKAP13 GDPD1 LYN SERPINB9 ATF6 SNAP23 IFI35 UBE2F SLC9A9 CASP4 IL16 SETX MEF2C GIMAP1 IFNAR1 PRAL KLHL5 TAB2 SERINC1 KDM1B TLN1 GBP2 RUBCN PLAAT4 ADD3 PATL1 GNGT2 CXCL11 ATP7A ISG15 NUB1 RNF213-AS1 TMEM140 GBP5 CASP7 LINC01232 IFIT6P VCL TAP2 MARF1 SH3GLB1 TLR1 TUT7 FCGR3A ABCC3 KSR1 STOM KLHL6 ATP6V1A FAM120AOS AIDA SPOP ITSN2 C10orf105 AC004466.2 RC3H2 VPS26C BTN3A2 ACSL5 NECAP2 FOXN3 PTP4A2 KLF3 ARHGAP18 XIAP AC104837.2 CDC40 GIMAP5 BCL2A1 GIMAP4 TCP11L2 MAPK1 MTMR12 RIPOR2 CLEC7A SAMD4A BIRC2 YIPF1 CASP5 WASF2 ARL6IP5 AC124319.3 GNA13 CD300A SMTNL1 ABHD2 CHURC1 IL15 CARD6 HDAC7 CTDSP2 RNF144B EFCAB14 CEMIP2 CORO1C TMEM229B UHMK1 HPSE PDCD1LG2 AC124319.1 CDC73 ARHGEF3 ELK3 MR1 GGPS1 ASB8 EXOC1 USP30-AS1 TOX4 CTR9 BACH1 AC113404.3 TRIP4 RAB11A CARD16 NCK1 ARRB1 WAC RNF20 CAB39 AL356056.2 ABI3 STK4 AC106712.1 SECTM1 MOV10 VRK2 IGF2BP3 GUCY1B1 SCARB2 CDS2 NEURL3 KIDINS220 BICRAL IL1B KCTD20 UBQLN2 Y_RNA SLC9A7 SECISBP2L JAK2 IFIT2 IFIT1 ZNF143 CCRL2 AL136295.5 SHFL MX2 BIN2 WNK1 FBXW11 ADAM10 IFI27 DOCK11 LAMTOR3 LRRC8D RABGAP1 SNAP29 NRBF2 C16orf74 PLCL2 AP3S2 NRIR MAT2B WDR44 STK38 KIAA0040 ST6GAL1 MTND4P23 ZNF24 BRAP SWAP70 LZIC BNIP3L APOBEC3F CHST7 TRANK1 RETREG3 BMP2K CCNDBP1 VAPB SUSD6 LCP2 LINC00847 LPIN2 LINC01547 CXCL16 TAB3 ZNFX1 CCNY CREBL2 FNIP2 RPS6KC1 ACSL4 BTN3A1 HCP5 C6orf62 FAM118B VTI1B SYNRG GRK3 TRMT1L PRKCB PLPPR3 FGD6 C11orf97 TNFRSF8 NCOA2 AC004466.1 PDCD10 MSNP1 CNOT6L LATS1 MSN COMMD2 ZNF845 PANX1 IQSEC1 PSMB8-AS1 TMBIM1 AHCYL1 FBXO8 KLHL12 TRIM25 APOBEC3D FMR1 STYX CTSS ZMYND15 CASP1 GNG10 DDI2 RIPK1 OGFRL1 CCR5 SIPA1L1 NCOA3 BIN2P1 AP2B1 SH3BGRL DIP2B RNF13 TESK2 SLK MOB3C TCEANC RDH11 GVINP1 CLIP4 HCFC2 PDCL GLTP AC083862.1 TSG101 SYNJ1 VNN2 RNF111 PCYT1A CDC42P3 RNF145 FOXO4 SLC18B1 RBBP5 MFSD11 NRIP1 CERT1 BROX TMLHE SEC23IP LINC01011 MYOM1 STK10 RNF141 ARL2BP NEK9 OSBPL11 ZNF710 TTC39B BIRC3 RAP1A LINC02432 MBNL2 AC069547.1 HELZ PICALM PTPRA PPP1R27 RECQL CARD17 PXN USP9X HERC3 STX6 AZU1 NMT1 SRC FEM1B TRAF6 DAPK1 LINC02085 DRAM2 PPP2R5C ACOX1 C3orf38 ZNF436 VEZF1 TMEM268 LYPD2 ITGB1 SP140L LSM14A WDR82 THAP2 FAM104A PNRC2 STX12 CHMP2B PTGS1 CRNKL1 ITM2B TBK1 ATMIN INPP4B GALNT10 AP3M1 GSKIP KBTBD11 JAML USP25 LPGAT1 ARHGAP30 PSME1 RASSF5 SGTB PARP11 LTA4H TMEM131 PHOSPHO1 RGP1 TMEM184C TRIM21 MS4A3 LINC00299 AC242426.2 IL15RA CALHM6 RAB10 KAT2B PDK3 HMGCS1 CD46 ACOT9 MFAP3 AC020931.1 ZNF217 HMOX1 DYNC1H1 RNF2 TRIM5 PEX12 MAP3K3 RESF1 CMKLR1 CLTC CCR5AS CPSF2 SP1 CHD8 AC124319.2 ZNF106 HERC1 IL27RA AC048337.1 PRDM4 AC135507.1 PCDH12 PPP2R5A ALKBH1 MYD88 UBR2 CUL4B SLFN5 AL844908.1 RABGAP1L AC004241.5 ILRUN ETV7-AS1 TDP2 C1orf131 MED1 P2RY12 C6orf89 GABPA CLPX MTPN MPZL3 KLHL9 TBC1D23 SPG11 AC004241.1 DPYD CRTAM TEP1 KIAA0513 MAPKAP1 DHX8 CCDC174 SNTB2 AL162424.1 R3HCC1L ANXA4 PLEKHO2 BTK JKAMP AC245884.12 RAB11FIP4 MPP1 MFAP1 EPS15 ERCC4 ATP11C PAPOLG TTC33 NSL1 STIM2 LTN1 SEMA4D RASA3 RASSF3 MAML2 FGD5-AS1 CYLD GPT2 SPTLC1 SERINC3 MBOAT1 SNAPC3 ABI1 PTTG1IP SLAIN2 PCGF5 ZNF267 AC004241.2 STAT5B TRIM44 OXSR1 CTCF TCF20 MAPRE2 LMBRD1 PIAS1 TNFAIP1 AC112229.3 SHISA5 DAPP1 ICAM2 BTBD10 WDR11 ZFP36L1 TNFSF10 SLAMF8 CGGBP1 EXOC8 UBLCP1 DCAF7 VAV1 SLC44A2 CRKL SLC37A3 CCDC69 PPP4R3B CREBRF SYT15 FPR3 ARAP2 MARCHF7 SMARCA5 STAT5A GTF2B AGGF1 CEPT1 DR1 TRIP12 AC034199.1 STAG2 AC010247.1 TNKS2 MICU1 FOXN3-AS1 EPHB2 WARS1 PDCD6IP ZBTB38 ATRX NLK STAU1 NCKAP1L RHOC IQGAP2 ZBTB24 ELOA GNAI3 LILRA5 PPP1R2P1 RAP1B CD300E SRD5A1 UBE4A PIK3R4 ALDH1A2 PRSS12 UBR3 CCDC127 TINF2 AFTPH SOS1 XXYLT1-AS2 GLG1 RMND5A FAF2 SYNE1 GOLGA5 F2R CYREN LYSMD2 HCCS SLC4A8 CD79B AC069528.2 HECA ARAP1 CDC5L UBXN7 PPP6C LGALS8 YME1L1 AC099343.3 TCF7L2 FAM8A1 PRKCQ CLIP1 CRTC3 AC099552.2 GIN1 NUMB MBNL3 DNASE1L1 TRIM34 STK40 DENND2D RPS6KA3 DUSP11 SWT1 TTBK2 ANGPT1 FMO5 BECN1 C12orf76 SLC25A10 NRDC SSH1 NIN DOCK8 PSMB8 PPP2R5E CAMK1D SCYL2 CHST15 CCDC117 VWA5A APPBP2 SLC37A1 LRRC57 LINC02384 HSCB VTA1 RAB44 HTR1F RYBP USP33 IL10RA LINC00487 SLC43A2 RBMXL1 MAML3 SDC3 KDM5A LILRB2 EPS8 UBE2R2 C5orf51 SLAMF6 RN7SL105P IRAG1 NOTCH2 GPR155 SLC7A7 AL031985.1 FGD1 TRIM56 BTBD10P1 API5 XKR7 CUL2 RRAGA GTF2H1 ACTR10 ZDHHC20 AC245884.11 TLR6 ANKRD13C HEG1 PAXIP1-AS2 FFAR2 CAP1 SUFU IL6ST MSR1 GNB4 BAG5 TJP2 SLC35A5 ZNF281 MED8 AGPAT3 HEIH GPR65 RAB39B CLEC1A ITCH C5orf22 ARL5A STRN DHX40 STX7 PARG AC124798.1 LINC01675 TRIM23 SLA IRF1 THRAP3 SDAD1 MMAA ABRAXAS2 LY96 PTPN11 UBL3 XIRP2 RAB9A FOLR2 PRKCH CANT1 DYNC1LI1 CARMIL1 CLEC4F SERPINB6 ATF1 ITPR1 PSEN1 VPS45 CMTR2 ICE1 TMCC1 SGMS1-AS1 BAZ2A TOP1 SH3TC1 SLC31A1 TRIM22 RPAP3 XIRP2-AS1 ZSCAN29 CITED4 HFE EIF2AK4 NEK7 FAM110A SPATA13 AC087277.2 PDIA3P1 PRKACB TXNDC16 AL645929.1 STXBP3 PKD2L1 ENOX2 HMG20A DHX29 MTHFR HMGCR TMEM131L REPS2 RGL1 BTBD1 CRNDE CNST YBX1P8 BCL2L14 TSC22D2 HSPE1P18 AC100803.4 TUG1 LIPC THAP12 CTSO GLE1 STAM2 NBPF10 A2M-AS1 MTND5P28 DPP8 AL032821.1 RPL5P13 RFX8 TIGD2 CLEC9A HLA-F DHFR2 AC004466.3 BCDIN3D PDPK1 TATDN3 RGS10 TPST2 VMO1 COPA AC016582.2 TMEM87B PIWIL4 GOSR2 SLC25A30 PSMF1 CD84 ARL14EP HNRNPH2 TTYH2 LINC00926 ARID5B ADD1 CD48 CCT8P1 ARHGAP9 EHBP1L1 ARHGAP27 RIC1 VWF PSMD14P1 RBSN GFI1 RO60 KAT6A MYLK CX3CR1 OLFM1 IFITM3 XRN1 ALDH1A1 RPL7AP64 MBP UBE4B CIR1 RCSD1 STX17 ZNF699 AC245884.2 RERE ZBTB34 DYRK1A ZNF2 VNN1 BANP PAPOLA SESN1 MAN1A2 SLC6A6 LINC01094 RAB22A AC100803.2 AQR SYNJ2BP TM9SF4 RNF149 UST VCPIP1 SNX2 TLR7 SLC4A1AP FOXO3 LINC01881 MSL3 RAB5A KPNA4 IKZF1 CDK19 DUSP7 KRBOX4 LIMS1-AS1 CCDC71L PRR5L ZNF490 EPC2 HMGB3 XPR1 LIG4 RNASEL LPIN1 AREL1 KTN1 CPNE3 DHRS4-AS1 SP3 USP6NL LINC02528 CDV3 CDH23 RALB AC089983.1 SEC24B PPP3CB-AS1 F2RL1 OXR1 RNF14 DDX23 UTRN SEPTIN9 AP000943.1 P2RX7 FAM168A FAM13A ZNF28 CAPZA2 PGAM1P7 RALBP1 KIF2A NCOA1 UBE2Q1 PPP4R3A IWS1 MAP3K14 DGLUCY WASHC4 RHOT1 KCTD18 ARID4B FCGR3B LILRP2 MAGT1 AC003070.1 TNRC6A CCDC6 CHAD SLC6A16 ARFIP1 RAB14 NPR3 PACS1 KLRG1 AL359073.1 VPS39 SCARF1 PDLIM5 PHF3 RNU6ATAC6P BATF3 PPID REST RNF6 SIGLEC11 HTT FBXO34 SMG7 SRBD1 OSBPL3 CWC22 OSBPL8 ZNF740 GID4 KIAA0232 CLEC2B TNFSF8 NPC1 ZNF821 CLCC1 EXOC3L4 AP4E1 USF3 ZFYVE26 RANBP2 MTFMT AC009163.7 STK24 CGRRF1 TMCC3 AP001972.5 SBF2 ARHGAP25 CD2AP | IFIH1 DTX3L PARP14 DDX60 OAS2 PARP9 SAMD9 EIF2AK2 DDX58 SAMD9L APOL3 APOL6 SP110 RTP4 OAS3 ADAR LINC01232 EPSTI1 STAT1 GBP4 IFI16 GIMAP2 GIMAP6 NMI APOBEC3G UBE2L6 USP38 ZNF845 DR1 BTN3A3 LAP3 MEF2C MX1 UHMK1 ACSL5 IRF2 CLCN3 CDC40 MTMR12 IFI44 RNF213 OAS1 PARP11 HERC6 ARL14EP AL353751.1 GBP3 TAP2 TRIM69 AC009950.1 SLC38A1 PLCL2 CDC73 TMEM268 PECAM1 ELK3 EFCAB14 PHF11 TMEM184C SLC12A6 GBP1 FOXN3 WIPF1 IFI44L RBM18 RO60 HCP5 SETX RAB3GAP1 SLK RNF145 ZNF217 GIMAP7 ZNF24 APOL1 KLHL5 SLC9A7 CNOT6L SGMS1 IFIT6P JAK1 THAP2 PANX1 TUT7 USP30-AS1 LIMS1 TRIM44 MMAA ATF7IP SP100 ST6GAL1 ATP6V1A N4BP1 VCL CASP10 LIPA PEX12 AC004551.1 GVINP1 ASB7 RBBP5 IKZF1 ZNF816 KLHL6 PSMB9 HERC3 SMARCA5 MAN1A2 SEC14L1 ZDHHC4P1 DUSP11 GUCY1B1 BIRC3 EXOC8 DCAF7 BROX VEZF1 WDR82 SLC25A30 CUL4B PIP4K2A SEC23IP HELZ USP18 ZBTB24 DAPP1 NSL1 TCEANC STYX RMND5A ARAP2 GGPS1 PTPRC HERC1 TRMT1L TAB2 ANKRD13A GBP1P1 SERPINB9 DOCK11 MAPK1 CHURC1 RNF144B TRAF6 LAMTOR3 MAT2B DDI2 ITPR1 PRKCB ABRAXAS2 LSM14A ATRX C6orf62 MARF1 ATP11C NBR1 AASDHPPT FEM1B RNF20 STX7 CPSF2 RC3H2 PSMB8-AS1 CTR9 ELOA KIDINS220 GDPD1 ERCC4 ARL5A AC113404.3 LATS1 API5 ZBTB6 AC242426.2 THRAP3 GTPBP8 SP1 STK4 APOL2 NUS1P1 AKAP11 ARRB1 MFSD6 TMLHE KDM1B PPP2R5E AC116348.3 SDAD1 SPOP CD2AP TAP1 LZIC AC004466.2 ANGEL1 AIDA NCOA3 CYLD BTBD10 AKAP7 MBNL3 MORC3 RBMXL1 C5orf22 LPGAT1 CWC22 ACTR3C REST STOM KLHL9 AZU1 VRK2 NAA50 CLCC1 RNF2 MR1 SWAP70 PAPOLG PTPN11 PIK3R4 BIRC2 STARD7 CD46 SYNE1 ATF1 AL445490.1 TIGD2 IGF2BP3 PNRC2 TIMM10B DDX60L KIAA0040 CRNKL1 KRBOX4 DCK WDR44 DHFR2 STRN ZNF468 ABHD2 EXOC1 HLA-DPA1 BTBD1 BBX CDC5L CCNY PLAAT4 RPAP3 XIAP CLPX HPS5 ZBTB38 IFIT1 ELP4 RPS6KA3 LYN FOXN3-AS1 PRKCH RSAD2 TOP1 LMO2 GTF2H1 RAB8B ICE1 FBXW11 TRIM25 TLR1 RANBP2 C5orf51 SGMS1-AS1 TRIM23 PPP4R3B ANXA4 LRRC8D ATF6 CASP7 ARMCX5-GPRASP2 UBQLN2 ZNF436 AC087277.2 RHBDD1 FAM120AOS TMEM135 MTMR1 ZNF143 CCDC117 CHMP5 RNF6 PLSCR1 FBXO30 AFTPH ZNF28 LINC01675 MED1 EIF2S1 CIITA ATP7A IDH3A GSKIP VTI1B MLH3 MSANTD4 KCTD20 STAM2 RAB22A KIF2A SERINC1 CD300A TCF20 TDP2 TATDN3 PDIK1L AQR PDCD10 USF3 PATL1 FAM104A KLHL12 ADD3 C1orf131 PARP12 AKAP13 SPTLC1 APOBEC3F DDX6 KMT2A CDS2 PHF3 ZNF267 XRN1 AP001610.2 GIMAP8 APPBP2 ARL2BP RNU7-61P RAB14 CFAP97 AP3M1 SNAPC3 GNA13 CD52 ZNF624 HIVEP3 RAB11A HLA-DPB1 GRK3 CMPK2 SLC25A10 IFIT3 IFIT2 ZNF101 UBXN7 TRIM34 PLPPR3 MAPRE2 TCP11L1 UBL3 SECISBP2L HMGCS1 LINC00847 CTSO ITSN2 KIAA1143 SIKE1 PPP1R27 LEMD3 TOX4 CNST MEF2C-AS1 FBXL3 FBXO45 LINC00299 CARMIL1 WNK1 QSER1 SYNRG SEPTIN11 RESF1 GIN1 FMR1 SFT2D2 PARG UTP25 NEDD1 VTA1 OR10AD1 COMMD2 GCFC2 C3orf38 MFSD11 VPS41 BAG5 UBASH3B NCK1 USP9X CLTC KDM5A CGGBP1 IL16 YBX1P8 IREB2 CSNK2A1 AHCYL1 TMEM131 RNA5SP296 Z95114.3 AL137222.1 CASP4 WASF2 C12orf4 SNX6 RFK FAM118B PRSS12 BTN3A2 F2R OASL CEPT1 RABGAP1L VWA5A GIMAP5 MFAP3 ANGPT1 BICRAL CD84 GTF2A1 SH3GLB1 HLA-DRB5 RAB39B DYNC1LI1 PTER ZNF501 FAM122A AGGF1 NEK1 WBP1L MARCHF7 PGAM1P7 IL15RA UBE4A PPP2R5A PDCL MOB1A AC133065.3 ATP8A1 LINC02863 RAP1A CMTR1 SRC STAR HTATIP2 NCOA2 TBC1D23 C2orf49 SNAP23 ATMIN CDC42P3 FAM117B SWT1 FAM168A LRBA MN1 AC104837.2 MBNL2 FMO5 HLA-DRA SERPINB6 ARID4B VCPIP1 ASB8 HDAC7 FGD6 CRNDE SOD2 MRPL50 SMG7 PIAS1 MFAP1 MRPL19 GABPA NSUN3 NUDT21 RGP1 GNL3L ACSL4 TCP11L2 JAK2 IPO7 SLFN5 DISP1 VPS13D CTDSP2 AREL1 BTN3A1 INPP5F ABHD10 RBM45 FMO7P XRCC5 CERT1 NCBP1 TNKS2 CMTR2 LRIF1 DYNC1H1 SUCLA2 MYCBP2 TRIM5 DCAF17 SYNJ2BP EPS15 KLF3 RSF1 WDR89 RGS10 TMEM87B ERCC6 PROM1 UBE2F SLC49A4 RPS6KC1 IFNAR1 PAPOLA THAP12 RIPOR2 TNRC6B ZNF281 MTND5P28 RECQL LINC01645 HCCS SMG8 ABI1 TRANK1 PPP1CB NSD1 DIP2B GBP5 CHD8 PPP2R5C SOS1 QRSL1 AL512343.2 GUCY1A1 KTN1 PXN CRKL PPID USP28 RNF169 POLR2M TTC33 NAB1 DDO ZNF619 STX6 PICALM FNIP2 SLC35A5 JKAMP GCSAM EIF2AK4 GOLPH3 PALB2 SPAST VAPB KPNB1 RCSD1 PTP4A2 STK24 PRR5L BRAP HMGCR PDPK1 DHX57 MED13 RAB10 CLIP4 ESCO1 CREB1 USP10 UBR2 KPNA3 RIOX2 WAC ELK4 PPP6C TXNDC16 CHM DOCK8 KIAA1109 VAV3 AC116348.1 UBR3 CUL5 AC009656.1 AC108448.2 IQGAP2 C11orf58 ITPKB OXR1 TRAFD1 GPRIN3 CIPC CNOT1 HLA-DRB1 ARCN1 PSME1 SHOC1 CCDC174 NUB1 CTCF DIS3 NPAT FAM13A NEK9 BFAR KPNA4 TDG CRYBG2 RN7SL684P PARP1 MS4A3 MAP3K7 F2RL1 ZNF836 HELZ2 NMT1 HLA-DOA PDCD6IP DHRS4-AS1 SCAF11 CD34 ZNF416 NIPBL ACTR10 G3BP2 FAM120A MSNP1 SEC24B BTN2A2 STIM2 AC243960.1 RBM12 H3C6 SLC18B1 LPIN1 QTRT2 POGK TBC1D5 TTBK2 PAXIP1-AS2 PPP1R8 ALKBH8 SLC30A1 TAB3 ZBTB1 DGLUCY SLC4A8 HSCB AL355916.2 RDH11 AC133065.1 RABGAP1 ZNF490 CSTF2T OTUD4 ZNF770 CALHM6 AC104083.1 HLA-DMB PPIL4 AC135507.1 RAD23B TBK1 LRRC8B VPS4B SP140L ORC3 ZNF766 NRIP1 REL HLA-DMA CUL2 BCDIN3D DRAM2 MTND4P23 SLC37A1 DPYD ZNF2 VPS45 PNPO PTPRA KIF1B EPRS1 IRF1-AS1 LIG4 ZSCAN29 PPP1CC ARMCX5 RAB44 STX17 WAPL GNAI3 HLA-E ANKRD13C ENG HACD2 TMEM123 MYLK AL359220.1 GIMAP4 SENP1 NDUFAF7 ZNF426 DAPK1 NEXN AP2B1 MOB1B MSN ZNF702P NIN SSH1 DHX8 CNOT11 PSIP1 NOL8 SLAMF6 ZSCAN16 CAMK1D STX12 HCFC2 LIPC LIN7A AC069547.1 CAB39 ANKRD17 METTL6 ASF1A AL356056.2 TRIP12 NFKB1 NOTCH2 TASOR KPNA1 ADAM10 KAT6A GDAP2 RAB29 IWS1 GNGT2 KDM4C GPT2 AC124319.1 CEP250-AS1 NFATC3 LY75 ENDOD1 ZFYVE26 GSK3B GIGYF2 DENND2D GLE1 PTGER4 TRIP11 AL157786.1 TLR3 KSR1 TMEM229B PUS10 Y_RNA ZNF557 TMEM140 ARHGAP25 ACBD3 LINC00926 ITCH SEL1L FBXO11 POLR2B SLA SP4 HNRNPR GNB4 SIPA1L1 NCKAP1L SNAPC5 FAF2 GIMAP1 IRF9 PRRC2C Y_RNA GTF2F2 AC005288.1 TMEM106B CDV3 GFM1 MIEF1 SPG11 RAB21 P2RY14 CTSG BBS10 HTR7P1 XXYLT1-AS2 MYOM1 DAAM1 RNASE3 NKAPD1 PA2G4P6 CTSS TTC5 CHST7 RAP1B NRBF2 ZNF146 LYSMD2 DUSP7 OPA1 TBX1 UTRN DNAJB4 PACS1 USP33 ZNF430 CFD SLF2 LARP4 RNF213-AS1 SPATA13 TIPRL HNRNPH1P1 AC012020.1 ITPKB-IT1 ACTR2 AL645929.1 CYB561 SLAIN2 RNF13 AL136114.1 PUS3 AP5M1 SP3 DBT ME2 TSEN15 LINC02100 UPF2 AC079203.2 KPNA6 CITED4 LACTB2-AS1 SRBD1 AL591623.1 CSTF1 IRAG2 AC009704.2 TEP1 LINC01719 C6orf89 POC1B RUBCN AP001962.1 DHX40 NEK6 ZNF148 C16orf74 A2M-AS1 R3HCC1L FAM30A CYTH4 TMEM260 TMEM19 SERPINI1 LTA4H PDIA3P1 CCRL2 ICE2 YIPF1 ZNF525 SMG1 OXSR1 FBXL4 PRR14L ZNF701 SLC12A7 AC023510.1 SBF2 BIN2 SLC5A3 ARHGEF3 SERINC3 VEZT AP001972.5 CEBPZ ZNF699 ZDHHC20 CEP85L PSMB8 DYRK2 PAK2 SLC9A9 APC WASHC4 ATG4C PTAR1 ZNF561 LPP CLEC2B SETD2 TMF1 AC023825.1 PLRG1 MACF1 VAV3-AS1 AC004241.2 C15orf40 VWA8 PPFIA1 RFX5 SNX29 VIRMA LTN1 PIGCP1 CA13 UFL1 SCAF8 MAP3K14 APOBEC3D FAM98B DICER1 PTPN1 HMG20A ZNF81 AC024257.3 ZNF260 ZFX RN7SL19P SNX19 PTENP1 ENOX2 AC100803.2 PPP3R1 STAG2 NHLRC2 AC099343.3 CHMP2B ZNF543 PPP2R1B ZNF740 IRX5 MMGT1 ACOX1 SYNJ1 TEKT2 SLC6A16 C21orf91 SESN1 GTF2E1 CD47 SGPL1 PRKACB MMRN1 ZFP90 MRPS14 ALKBH1 NUP133 ITM2B RFX7 ZNF106 KIAA0232 CBLL1 FUNDC2P4 FAM120B CRLF3 ZNF322 EED AC020904.2 GOSR2 ARL6IP5 TLR6 VPS33A TPR CPT1A MOSMO XRN2 XPO4 OGFRL1 NPC1 ITGAL AP3S2 HS2ST1 MTFMT AGPAT3 SMU1 TM2D2 ASXL2 CCR5 WDR11 VPS50 UBE4B | PARP9 TRIM69 OAS1 IFIH1 MX1 AC004551.1 SAMD9L STAT1 EPSTI1 OAS3 DDX58 OASL OAS2 PARP14 GIMAP7 GBP1 IFI44 APOL6 APOL3 RNF213 SP110 GIMAP8 DTX3L AP001610.2 SAMD9 GIMAP6 IRF2 TRAFD1 LAP3 GIMAP4 IFI44L SOD2 GIMAP2 TLR1 PARP12 RAB8B DDX60 ZDHHC4P1 AL445490.1 AKAP13 APOL2 SGMS1 GIMAP1 ADAR PTPRC NBR1 GIMAP5 N4BP1 AC009950.1 IRF9 PHF11 CASP10 Z95114.3 APOL1 TUT7 SLC9A9 BTN3A3 TAP1 EIF2AK2 HELZ2 PLSCR1 KLF3 IFI16 RNF213-AS1 CCR5 HLA-E GBP3 UBE2L6 JAK2 CTSS JAK1 SETX LIPA CLCN3 GBP5 SERPINB9 RIPOR2 RUBCN SIPA1L1 PECAM1 ADD3 SEC14L1 IFNAR1 SNX6 LYN DDX60L IL16 TCP11L2 NRBF2 NCOA3 OGFRL1 WIPF1 STX12 ZNF143 ARHGEF3 AP3S2 TAB2 SP100 ANKRD13A ATP6V1A SRC IFIT1 SLFN5 SERINC1 APOBEC3G GNGT2 PXN RNF144B PSMB9 KSR1 WBP1L BIN2 PICALM SNAP23 GBP4 HERC6 MORC3 AL356056.2 AC124319.1 RESF1 BIRC3 STK4 CHMP5 LTA4H KDM1B PIP4K2A TLR6 XIAP KLHL5 RSAD2 SLC18B1 ZNF217 PLCL2 MARF1 ITSN2 RNF13 FGD6 AIDA PATL1 HCP5 DPYD CASP4 CERT1 FAM120AOS LIMS1 AC069547.1 DRAM2 EFCAB14 AC113404.3 HERC3 AC004466.2 MAPK1 SYNJ1 TMEM140 LPGAT1 PTP4A2 CCRL2 FAM118B KIAA0040 UBE2F RTP4 PSMB8-AS1 BTN3A1 TAP2 PDPK1 CMPK2 CTDSP2 RABGAP1L ZBTB38 SBF2 ASB7 RAP1A KIDINS220 CAB39 PPP2R5C ZNF106 MTMR12 CD300A CCNY BICRAL ITM2B FNIP2 GBP1P1 STX7 PIAS1 GVINP1 DAPP1 LINC01232 ATP7A NOTCH2 YIPF1 WASF2 CLIP4 ST6GAL1 USP18 GNA13 TMEM268 UHMK1 MR1 RAP1B GRK3 AL353751.1 HCFC2 ADAM10 PPP2R5A SLC12A6 RECQL FMR1 ABHD2 WASHC4 UBR2 RAB10 MFSD11 MTND4P23 BROX KLHL6 CDC40 CAMK1D NCOA2 CD46 EXOC1 WNK1 SYNRG CDC73 PRKCB RAB3GAP1 VCL ZNF845 DOCK8 CEPT1 Y_RNA GNAI3 NCK1 FAM13A MEF2C PPP1R27 LATS1 ZDHHC20 DOCK11 RAB22A BTBD10 SLA WDR44 TRIM25 SPG11 ACSL4 TNKS2 SH3GLB1 NPC1 STX6 RAB11A IGF2BP3 MSNP1 GDPD1 UTRN LAMTOR3 RABGAP1 TATDN3 IFIT6P CHST7 CHURC1 TRIM34 NIN GIN1 CDS2 ZNF267 CARMIL1 AFTPH SPATA13 SECISBP2L MAP3K14 RC3H2 KIAA0232 RNF20 GPT2 RNF145 MBNL2 VTI1B R3HCC1L SYNE1 RBM18 BIRC2 HELZ ARRB1 IQGAP2 RDH11 UBE4A ZNF699 NSL1 ITCH CDC42P3 ACOX1 CLCC1 NUB1 LRRC8D CTSO SLC25A10 WAC NMI MSN ABI1 SLC4A8 TMEM131 SNAPC3 PRKCH EXOC8 ARL6IP5 AC100803.2 SPTLC1 C6orf62 HCCS MAT2B ATF6 STOM SP3 TCEANC KDM5A ARHGAP25 UBR3 CHMP2B ZNF436 SSH1 BTN3A2 UBQLN2 ELK3 VAPB RPS6KA3 ALKBH1 TOX4 SLAIN2 FOXN3-AS1 C6orf89 ANXA4 STYX SWT1 ZNF24 PAPOLG DENND2D AGPAT3 MMAA PANX1 CYLD TMEM229B DAPK1 GSKIP CASP7 HERC1 XRN1 DYNC1LI1 ARAP2 UBL3 GUCY1B1 WDR11 ERCC4 SP1 STAM2 CTR9 HDAC7 TEP1 TAB3 KCTD20 TBC1D23 LYSMD2 SLC25A30 USP38 TRIM23 DDI2 PLAAT4 RBBP5 RPS6KC1 MFAP3 VCPIP1 STK24 ELOA NMT1 ITPR1 BRAP TMLHE SLC9A7 JKAMP STIM2 CMTR1 LINC00299 EPS15 IL15RA TCF20 PARP11 PDCD10 TRMT1L SPOP FOXN3 CUL4B DR1 USP33 PDCD6IP OXSR1 YBX1P8 IKZF1 PLPPR3 FEM1B MYOM1 MARCHF7 GOSR2 CLTC |
